# The novel NADPH oxidase 4 selective inhibitor GLX7013114 counteracts human islet cell death *in vitro*

**DOI:** 10.1371/journal.pone.0204271

**Published:** 2018-09-28

**Authors:** Xuan Wang, Andris Elksnis, Per Wikström, Erik Walum, Nils Welsh, Per-Ola Carlsson

**Affiliations:** 1 Science for Life Laboratory, Department of Medical Cell Biology, Uppsala University, Uppsala, Sweden; 2 Glucox Biotech AB, Stockholm, Sweden; Maastricht University, NETHERLANDS

## Abstract

It has been proposed that pancreatic beta-cell dysfunction in type 2 diabetes is promoted by oxidative stress caused by NADPH oxidase (Nox) over-activity. The aim of the present study was to evaluate the efficacy of novel Nox inhibitors as protective agents against cytokine- or high glucose + palmitate-induced human beta-cell death. The Nox2 protein was present mainly in the cytoplasm and was induced by cytokines. Nox4 protein immunoreactivity, with some nuclear accumulation, was observed in human islet cells, and was not affected by islet culture in the presence of cytokines or high glucose + palmitate. Nox inhibitors with partial or no isoform selectivity (DPI, dapsone, GLX351322, and GLX481372) all reduced ROS production of human islet cells exposed to high glucose + palmitate. This was paralleled by improved viability and reduced caspase 3 activation. The Nox1 selective inhibitor ML171 failed to reduce human islet cell death in response to both cytokines and high glucose + palmitate. The selective Nox2 inhibitor Phox-I2 also failed to protect against cytokines, but protected partially against high glucose + palmitate-induced cellular death. The highly selective Nox4 inhibitor GLX7013114 protected islet cells against both cytokines and high glucose + palmitate. However, as no osmotic control for high glucose was used, we cannot exclude the possibility that the high glucose effect was due to osmosis. It is concluded that Nox4 may participate in stress-induced islet cell death in human islets *in vitro*. We propose that Nox4 mediates pro-apoptotic effects in intact islets under stressful conditions and that selective Nox4-inhibition may be a therapeutic strategy in type 2 diabetes.

## Introduction

Pancreatic islet overproduction of reactive oxygen species (ROS) may result in beta-cell failure and type 2 diabetes mellitus (T2DM) [1–4]. The excessive production and accumulation of ROS is, at least in part, due to hyperactivity of the NADPH oxidases (Nox). The Nox family consists of seven isoforms (Nox1-5 and DUOX1-2), which perform normal cellular functions at basal conditions, but when persistently activated produce harmful levels of ROS. Hyperactivity of some of the isoforms has been found to be an important driver in a number of diseases including diabetes and diabetes complications [5]. Both rat and human pancreatic beta-cells have been reported to express some of the Nox subunits [6–8], and *in vivo* studies have reported increased islet Nox-mediated ROS generation in diabetic rat and human islets, and that this was associated with reduced beta-cell function [9].

Pharmacological Nox inhibitors have previously been administered both in vitro and in vivo to evaluate the putative role of Nox enzymes in different pathological processes, such as glucose intolerance and beta-cell dysfunction. Unfortunately, some of these Nox inhibitors, such as apocynin and diphenylene iodonium, are today considered not to be selective Nox inhibitors. Instead, novel Nox inhibitors with better Nox and Nox isoform specificity have been developed [10]. Examples of such Nox inhibitors are ML171, which selectively inhibits Nox1 [11], GLX351322, which targets Nox4 preferentially over Nox2 [12], and the Nox2 inhibitors Phox-I2 [13] and GSK2795039 [14]. In a recent study using the Nox4 selective inhibitor GLX351322, we observed amelioration of high-fat diet-induced glucose intolerance [12]. In addition, inhibition of also Nox1 and Nox2 has been suggested to improve beta-cell function when exposed to diabetic conditions and inflammatory cytokines [15,16].

Specificity of inhibitors for different Nox isoforms will be important in the development of drugs, minimizing their side effects. We presently report the generation of a new Nox inhibitor, GLX7013114, with improved pharmacological characteristics when it comes to efficacy and specificity in the inhibition of Nox4. Using a variety of Nox inhibitors, including this Nox4 inhibitor, we tested the possibility to protect against pro-inflammatory cytokine- or high glucose + palmitate-induced human islet cell death *in vitro*. These models are useful in the study of T2DM as pro-inflammatory cytokines and the combination of high glucose + palmitate both promote beta-cell dysfunction and death *in vitro* [17,18], and are considered to participate in the pathogenesis of T2DM [19,20].

## Methods

### Chemicals and cells used in the development and characterization of Nox4 inhibitors

RPMI 1640 with Glutamax, DMEM/F12 (1:1), Hanks' buffered salt solution (HBSS), fetal bovine serum (FBS), and Amplex red were purchased from Invitrogen, Paisley, UK. Pest (penicillin, streptomycin), neomycin, ionomycin, phorbolmyristateacetate (PMA), diphenyleneiodoniumchloride (DPI), dapsone, ML-171, Phox-I2, xanthine, hypoxanthine, xanthine oxidase, DMSO, DPPH (2,2-diphenyl-1-1picrylhydrazyl), Tween20, Sucrose, flavin adenine dinucleotide (FAD), Phosphatidic acid, ethylene glycol-bis(β-aminoethyl ether)-N,N,N',N'-tetraacetic acid (EGTA), horseradish peroxidase (HRP) and NADPH were purchased from Sigma–Aldrich. HEK293 overexpressing Nox4 (CJ Nox4) cells were purchased from Redoxis, Lund, Sweden. HEK 293 cells expressing Nox5 and CHO cells expressing Nox1 were a kind gift from the Vincent Jaque Center Medical Universitaire, Geneva, Switzerland. GKT136901, a Nox1/Nox4 selective inhibitor, was a kind gift from prof. Harald HH Schmidt (Maastricht University, Netherlands).

### Identification of Nox4 inhibitors in a high-throughput screen (HTS)

Primary identification from 40 000 chemicals were performed by using T-Rex-293 cells with inducible Nox4 overexpression in a 384-well format HTS Amplex red based assay. A threshold of 50% inhibition of Nox4 activity identified more than 700 hits. A counter screen with 4 μM hydrogen peroxide reduced false positive down to 90 structurally diverse compounds. These were further investigated in dose-response curves and 54 compounds received an IC_50_ with the most potent below 1 μM.

### Redox assay

DPPH (2,2-diphenyl-1-1picrylhydrazyl), a well-known sensitive chemical of monitoring reactions involving radicals [21], was used as control to exclude any redox active compound.

### Determination of GLX inhibitor IC_50_ for Nox2 inhibition in human neutrophils

Selected hits from the dose-response curves originating from high-throughput screen campaign as well as structure activity relationship-(SAR)-developed GLX inhibitors were tested for selectivity against Nox2 in isolated neutrophils from whole blood (human), as previously described [12].

### SAR development to receive GLX481372 and GLX7013114

The best hits from the Nox4 over Nox2 selection were further developed in a SAR mode with medicinal chemists, Nox isoform assays (membrane and whole cell) and pharmacological evaluation. The SAR campaign resulted into selection of GLX481372 (S1 Fig) and GLX7013114.

### Determination of activity and selectivity of GLX481372 and GLX7013114 in selection assays of intact Nox expressing HEK and CHO cells

Adherent cells (CHO, HEK) were collected by trypsinization, centrifuged, washed with HBSS, counted, and resuspended in HBSS. Cells were seeded in 96-well black flat-bottom plates at a density of 50,000–100,000 cells/well. All compounds were dissolved in DMSO and concentrations ranging from 0.003 to 200 μM were tested in Nox cellular assays with a final concentration of DMSO of 1%. Cells were incubated at 37° C with the compounds for 30 min before measurement. Cells expressing Nox1 and Nox2 were activated with the PKC activator PMA (0.1 μM). Nox5 was activated with the Ca^2+^ ionophore ionomycin (1 μM) and further enforced with PMA. The CJ HEK 293 cells overexpressed Nox4 constitutively. In the HTS screen HEK 293 TRex was used and tetracycline (1 mg/ml) was added 18h before measurement to induce Nox4 expression. Production of hydrogen peroxide by Nox in intact cells was measured using Amplex red fluorescence as described [22].

### ROS measurement in Nox4 membrane assay

Nox4-containing membranes were produced by trypsination to detach 10^7^ HEK 293 cells overexpressing Nox4. Cells were then suspended and homogenized in 1.5 ml of a sonication buffer containing PBS, sucrose (11%), and EGTA (1 mM) supplemented with protease inhibitors (Sigma, P8340) and processed as described [22]. Measurement of ROS generation by Nox4-containing membranes was performed by Amplex Red assay in black 96-well flat-bottom plates. Assay reagents including HRP, FAD, Phosphatidic acid and Amplex red were added and then NADPH to initiate production of hydrogen peroxide.

### Amplex red xanthine oxidase (XO) assay

The assay was designed for Amplex red analysis of production of hydrogen peroxide [14]. Test compounds were incubated with 5 mU/ml bovine derived xanthine oxidase for 15 min at room temperature and followed by the addition of substrate and detection mix (final concentrations of 0.2 U/ml HRP, 5 μM hypoxanthine, and 50 μM Amplex Red).

### Pharmacological methods

#### Determination of solubility

Solubility of solid GLX compounds was tested utilizing the shake flask method in 0.1 M phosphate buffer at a pH 7.4. Briefly 0.5 mg of GLX substance and 0.5 ml phosphate buffer in HPLC glass vials were sealed and incubated for 24h under rotation (900 rpm) at 37°C. After the incubation an aliquot was transferred to conical glass inserts and centrifuged for 20 min at 10,000 x g and the supernatant was analyzed with liquid chromatography-mass spectrometry/mass spectrometry (LC-MS/MS).

Kinetic solubility was determined in a similar way: 2 μl test compound (from 10 mM DMSO stock) was diluted 100x in 10 mM potassium phosphate pH 7.4 in a HPLC glass vial, sealed and incubated for 24h under rotation (900 rpm) at room temperature. After the incubation 150 μl was transferred to conical glass inserts and centrifuged for 20 min at 10,000 x g. Two μl of the supernatant was transferred to a 96-well plate, diluted 100x with acetonitrile/H_2_O (60/40, vol/vol) and analyzed by LC-MS/MS.

#### Determination of chemical stability

GLX inhibitor was pipetted into an HPLC vial, from 10 mM DMSO, to yield 2 μM final concentration in three separate vials containing buffers with different pH. At reaction start the three different buffers were mixed with isopropanol (1:2, buffer:isopropanol). The buffers used were: pH 2 (H_3_PO_4_/KH_2_PO_4_ 10 mM), pH 7.4 (KH_2_PO_4_/K_2_HPO_4_ 10 mM) and pH 10 (Glycine/NaOH 10 mM). Immediately (<1 minute) after buffer or buffer/isopropanol addition, an 100 μl aliquot was added to a separate plate containing 100 μl acetonitrile: H_2_O (60:40) and Warfarin (internal standard, IS), sealed and frozen at -20°C. This test was made for 2h and 20h. Analysis was performed on a XEVO TQ mass spectrometer coupled to an Acquity UPLC system in ESI+MRM mode, separation on a BEH C18 2 x 50 mm column.

#### Determination of metabolic stability

The microsomal metabolic stability assay utilizes pooled human, or animal (mouse) species, liver microsomes with supplemented cofactor (NADPH) to primarily facilitate cytochrome P450 (CYP) reactivity against target compound. Target compound (1 μM incubation concentration) and microsomes (0.5 mg/ml incubation concentration) were diluted in 0.1 M phosphate buffer, pH 7.4, in a volume of 150 μl. The reaction was initiated with addition of NADPH (1 mM). The incubation times were 0, 5, 15, 40 min and the reaction was quenched, at each time point, by addition of 100 μl acetonitrile containing Warfarin as IS. The plate was then sealed, centrifuged and frozen at -20 C until LC-MS/MS analysis.

#### Determination of GLX plasma protein binding and stability

GLX inhibitors were incubated at 10 μM in plasma and then equilibrium dialysed, using a rapid equilibrium device, for 4h. Protein binding (fu) value and remaining inhibitor after 4h was determined by LS/MS as previously described [12].

#### Determination of GLX inhibitor transport

Caco-2 membrane permeability was performed as described in detail previously [23]. In brief, Caco-2 cell monolayers were grown on permeable filter support and used for transport study on day 21 after seeding. Prior to the experiment GLX inhibitor solution (10 μM) was prepared and warmed to 37°C. The Caco-2 filters were washed with pre warmed HBSS prior to the experiment, and thereafter the experiment was started by applying the donor solution on the apical side or basolateral side, depending on which direction that was monitored. The transport experiments were carried out at pH 7.4 in both the apical and basolateral chambers. The experiments were performed at 37°C and with a stirring rate of 500 rpm. The receiver compartment was sampled at 15, 30, and 60 min, and at 60 min also a final sample from the donor chamber was taken in order to calculate the mass balance of the compound. Directly after the termination of the experiment the filter inserts were washed with pre-warmed HBSS and the membrane integrity was checked. This was performed by trans-epithelial electrical resistance (TEER) measurement. The experiment was validated by inclusion of the para-cellular marker ^14^C-mannitol and monitoring its permeability during the experiments.

#### Determination of i.p. pharmacokinetics in mouse (GLX7013114 and GLX481372)

The animal experiments were approved by the local ethical committee Malmö/Lund. Nox inhibitor GLX7013114 was administrated intraperitoneally in C57BL/6 female mice at hour 0 and blood was collected at termination of the animals at different time points post administration, i.e. 10 min, 30 min, 60 min, 90 min and 120 min, and plasma was prepared. 50 μl plasma was precipitated by adding 150 μl methanol containing Warfarin as IS. The sample plate was sealed, centrifuged and the supernatant analyzed by LC-MS/MS. LC-MS/MS analysis was performed on a Sciex QTRAP 6500 coupled to a Waters Acquity UPLC. Column HSS T3 50 x 2 mm. Mobile phase A: 0.1% formic acid, B: 0.1% formic acid in acetonitrile. Gradient between 95% A to 5% A over a 2.5 min analysis time.

### Human islets and EndoC-βH1 cell culture

Human pancreatic islets were kindly provided by Nordic Network for Clinical Islet Transplantation. Only organ donors who had agreed to donate for scientific purposes were included. Informed written consent to donate organs for medical and research purposes was obtained from donors, or relatives of donors, by the National Board of Health and Welfare (Socialstyrelsen), Sweden. Permission to obtain pancreatic islet tissue from the Nordic Network for Clinical Islet Transplantation was reviewed and approved by the local ethics committee (Regionala etikprövningsnämnden, Uppsala) in Uppsala, Sweden.

After isolation, the islets were cultured free-floating in Sterilin dishes in CMRL 1066 medium (ICN Biomedicals, Costa Mesa, CA, USA) containing 5.6 mM glucose, 10% fetal calf serum and 2 mM L-glutamine for 1–5 days. All cells were kept at 37°C in a humidified atmosphere with 5% CO_2_. Human EndoC-βH1 cells were cultured in the presence of 2% fatty acid free bovine serum albumin as previously described [24].

### ROS production in human islets

Human islets were incubated at control condition (cultured as described above) or with the addition of 1.5 mM palmitate (2% BSA) + 20 mM high glucose for 24h. During the last 3h of the 24h incubation, Nox inhibitors were added to the culture medium. During the last 40 min of the 24h incubation, human islets were loaded with the free radical indicator carboxy-H_2_DCFDA (5-(and-6)-carboxy-2',7'-dichlorodihydrofluorescein diacetate, 10 μM, Life Technologies, Stockholm, Sweden) at 37 ^o^C. Hoechst stain (Life Technologies), which labels cell nuclei, was added during the last 10 min of this incubation. The islets were then washed and placed on the stage of an inverted confocal microscope (Nikon TE2000-U) and analyzed for green (DCF—dichlorofluorescein) and blue fluorescence (Hoechst). For each treatment group, 5 images (100–250 cells in each image) were taken at different areas. The intensity of green (DCF) and blue (Hoechst) signals was quantified using Image J software (National Institutes of Health, USA) and ratios between green and blue signals were calculated as a relative measure of oxidative stress.

### Total ROS and mitochondrial superoxide production in EndoC-βH1 cells

EndoC-βH1 cells were loaded with H_2_DCFDA (Life Technologies), 10 μM, for total ROS production, and 5 μM MitoSOX Red (Invitrogen, Paisley, UK), for mitochondrial superoxide production, at 37 ^o^C for 30 min. The cells were then incubated with the Nox4 specific inhibitor GLX7013114 for 60 min. After trypsinization and washing, the DCF (total ROS, 530 nm) and MitoSOX (mitoROS, 580 nm) fluorescence intensities were quantified by flow cytometry.

### Immunofluorescence analysis

Staining of human islets for Nox2, Nox4 and cleaved caspase 3 was performed on cryosections (8 μm). EndoC-βH1 cells were cultured on coverslips before staining. The primary antibodies (rabbit anti-human Nox4 (1:300; NOVUS Biologics, Abingdon Oxon, UK), mouse anti-human Nox2 (1:250, Abcam, Cambridge, UK) and rabbit anti-human cleaved caspase 3 (1:300; Cell Signaling, Leiden, The Netherlands) were added at 4°C overnight. The secondary antibodies (highly cross-adsorbed Alexa Fluor 555 (1:500; Life Technologies) and Alexa Fluor 488 (1:300, Life Technologies) were added for 1 hour at room temperature. The nuclei were stained with Hoechst (1:10 000; Life Technologies). All images were obtained using the laser scanning confocal microscope Zeiss LSM 780.

### Western blot analysis

Cells were washed in cold PBS and lysed on ice in SDS-sample buffer (2% SDS, 0.15 M Tris, pH 6.8, 10% glycerol, 5% β-mercaptoethanol, bromphenol blue and Halt Phosphatase Inhibitor Cocktail, Thermo Fisher Scientific, Waltham, MA, USA). The samples were boiled for 5 min and separated on 10% SDS-PAGE gels. Proteins were transferred to LF-PVDF membranes (Merck, Darmstadt, Germany). The membranes were pre-blocked in 2.5% BSA for 1h and then incubated with anti-Nox4 (1:500, NOVUS), Nox2 (1:1000, Abcam), ERK1/2 (1:200, Santa Cruz Biotechnology, Santa Cruz, CA, USA) antibodies over-night at 4°C. Fluorescent anti-mouse/rabbit secondary antibodies were from Abcam. The bound antibodies were visualized with a LI-COR Odyssey Fc system (LI-Cor Biosciences, Lincoln, USA).

### Evaluation of human islet cell viability

The cell viability of human islet cells was assessed after culture with IL-1β (Peprotech, Rocky Hill, NJ, USA) (20 ng/ml) + IFN-γ (Peprotech) (20 ng/ml) or with 20 mM glucose + 1.5 mM sodium palmitate (2% fatty acid free albumin) for 24h or 72h. Cell viability was measured by staining cells with propidium iodide (20 μg/ml) and bisbenzimide (5 μg/ml) for 20 min at 37°C. The medium was replaced with PBS and the red and blue fluorescence was detected using a Nikon Eclipse fluorescence TE2000-U microscope (Nikon Instruments, Amsterdam, the Netherlands). The ratio of red to blue was taken as a relative measure of cell death (necrosis and late apoptosis) and was quantified using the Image J software (National Institutes of Health).

### Evaluation of EndoC-βH1 cell viability

The cell viability of EndoC-βH1 cells was assessed after culture with IL-1β (20 ng/ml) + IFN-γ (20 ng/ml) or with 20 mM glucose + 1.5 mM sodium palmitate (2% fatty acid free albumin) for 48h with or without Nox inhibitors. Cells were then vital stained with 10 μg/ml propidium iodide (PI) for 10 min at 37°C. Free-floating cells and cells attached to the culture dish were washed separately with PBS, pooled and examined by flow cytometry using a FACSCalibur (BD Biosciences, Franklin Lakes, NJ, USA). In each experimental group 10000 cells were examined and gated by PI intensity and FSC signal. The percentage of dead cells was analyzed using CELLQUEST software (BD Biosciences).

### Statistics

Results are shown as means ± standard error of the mean (S.E.M). Groups were compared with control using repeated measurement one-way ANOVA followed by Fisher PLD test. Two groups were compared using Student’s paired t-test.

## Results

### Characterization of the novel Nox4 inhibitors GLX481372 and GLX7013114

The results of the characterization of the novel Nox inhibitor GLX481372 (S1 Fig) are given in S1 Table. We observed that this inhibitor selectively targets Nox4 and Nox5 with IC_50_s for both enzymes in the sub-micromolar range. GLX7013114, on the other hand, inhibited only NOX4 at concentrations below 1 μM (S2 and S3 Figs, S2 Table). In these experiments GX7013114 was compared to GKT136901, a well known Nox1 and Nox4 selective inhibitor [10], and DPI, a non-selective Nox inhibitor [25]. GLX7013114 did not display any redox activity, as assessed by the DPPH assay (S4 Fig). GLX7013114 also showed total inactivity for inhibition of Xanthine oxidase (S5 Fig) as well as for Glucose oxidase (S2 Table). The different cell types used for IC_50_ analysis for Nox1-5 are described in S3 Table.

### Nox2 and Nox4 protein expression in human islets and EndoC- βH1 cells

**Fig 1:**
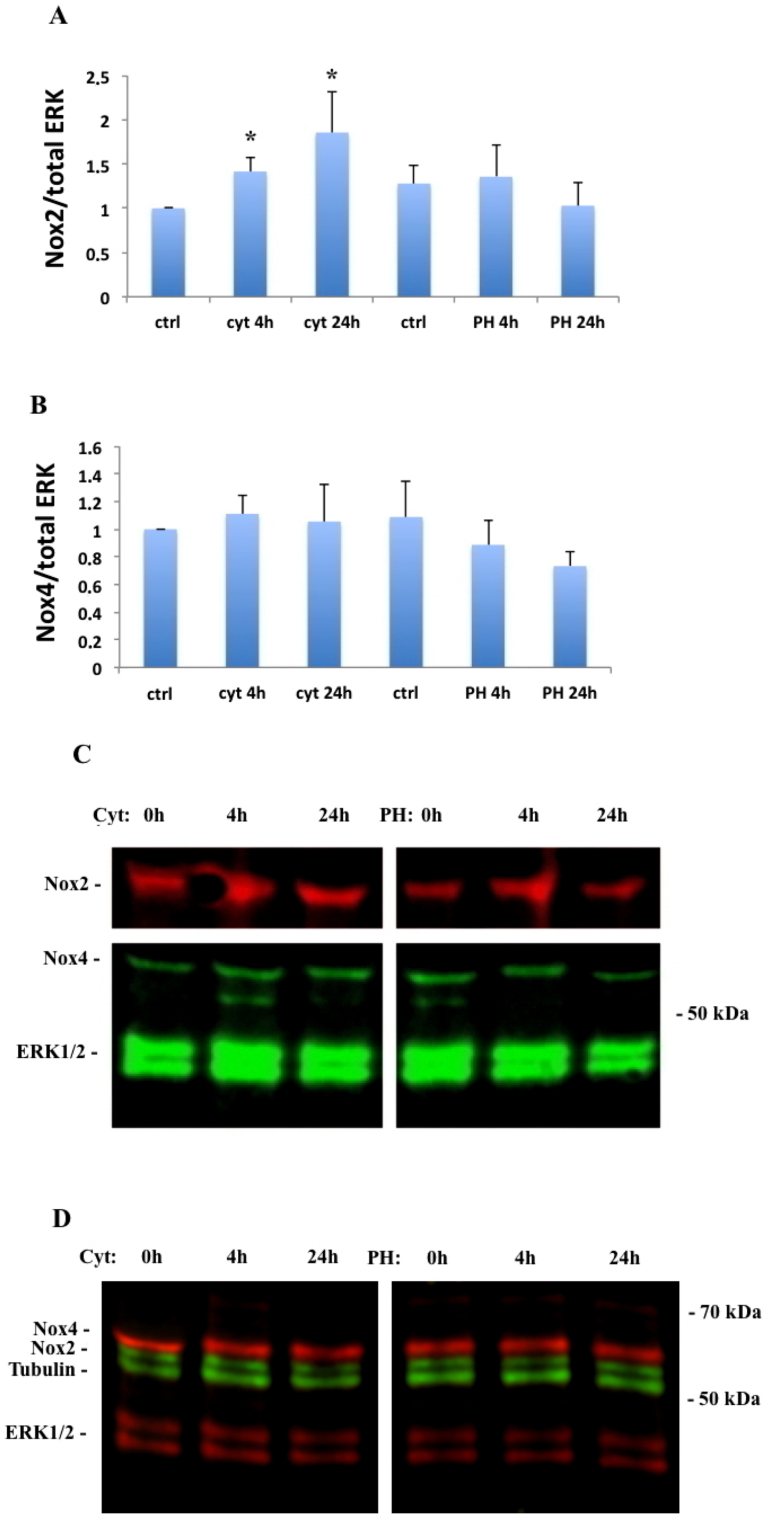
**Immunoblot analysis of Nox2 and Nox4 in human islets (A-C) and EndoC-**β**H1 cells (D).** Nox2 (A) and Nox4 (B) expression was assessed after 4h and 24h of IL-1β (20 ng/ml) + IFN-γ (20 ng/ml) (cyt) and palmitate (1.5 mM in 2% BSA) + high glucose (20 mM) (PH) exposure. The expression of Nox2 and Nox4 was normalized to total ERK, which was used as a loading control. Results are means ± S.E.M for 5 independent experiments. * denotes P<0.05 using Student’s paired t-test. (C) Representative immunoblot image for Nox2, Nox4 and total ERK in human islet cells. (D) Representative immunoblot image for Nox2 (green), Nox4 (red), tubulin (green) and total ERK (red) in EndoC-βH1 cells.

**Fig 2:**
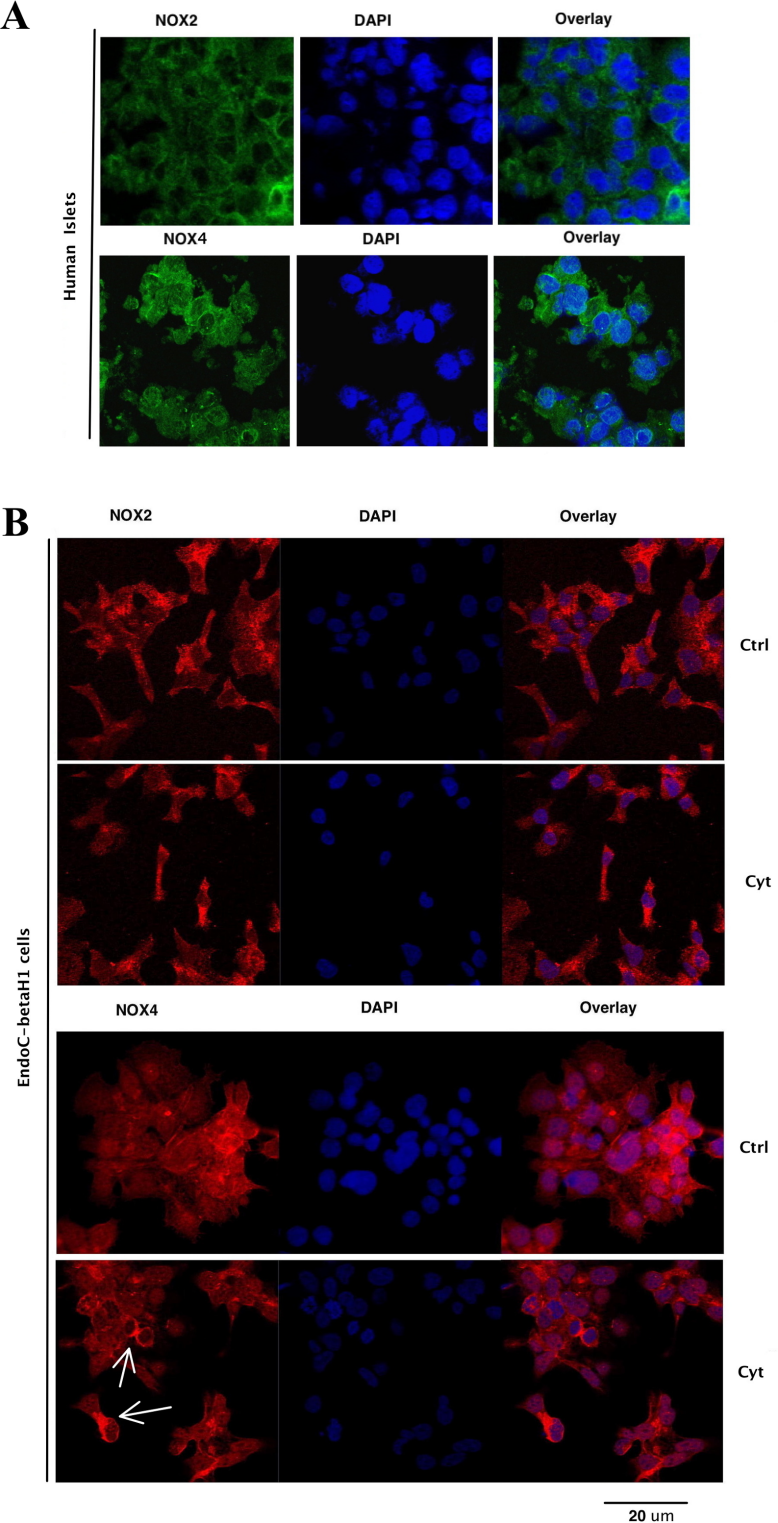
Immunofluorescence analysis of Nox2 and Nox4 expression in human islet cells and EndoC-βH1 cells. Islets and EndoC-**β**H1 cells were analyzed for Nox2/Nox4 immunofluorescence and counterstained with DAPI. Some cells were incubated for 24h with IL-1**β** (20 ng/ml) + IFN-**γ** (20 ng/ml) (cyt). Arrows point to EndoC-**β**H1 cells in late telophase with increased cytoplasmic Nox4 accumulation. Results are representative for 3 independent experiments.

Using the immunoblotting technique, both Nox2 and Nox4 immunoreactivity was observed in human islets (Fig 1A–1C). Cytokine (IL-1β and IFN-γ) incubation increased Nox2 expression at 4h and 24h, while palmitate + high glucose did not affect Nox2 levels (Fig 1A). No changes of Nox4 protein levels in islets were observed after cytokine or palmitate + high glucose treatment (Fig 1B). Nox2 and Nox4 immunoreactivity was observed also in EndoC- βH1 cells, but did not change in response to cytokine- or high glucose + palmitate exposure (Fig 1D). The Nox2 protein, as assessed by confocal immunofluorescence analysis, was present mainly in the cytoplasm of the human islet cells (Fig 2A). Also Nox4 protein immunoreactivity, with some nuclear membrane accumulation, was observed in human islet cells (Fig 2A). Both Nox2 and Nox4 immunoreactivity could be detected in a pure human beta-cell population, the EndoC- βH1 cells, in a similar expression pattern as that observed in human islet cells (Fig 2B). Culture of EndoC- βH1 cells in the presence of the cytokines IL-1 β + IFN-γ did not result in any obvious relocalization of either Nox2 or Nox4. Interestingly, EndoC-βH1 cells undergoing late stages of mitosis exhibited an increased cytoplasmic Nox4 accumulation (Fig 2B).

### Non-selective Nox inhibitors reduced high glucose + palmitate-induced ROS production and cell death in human islets

**Fig 3:**
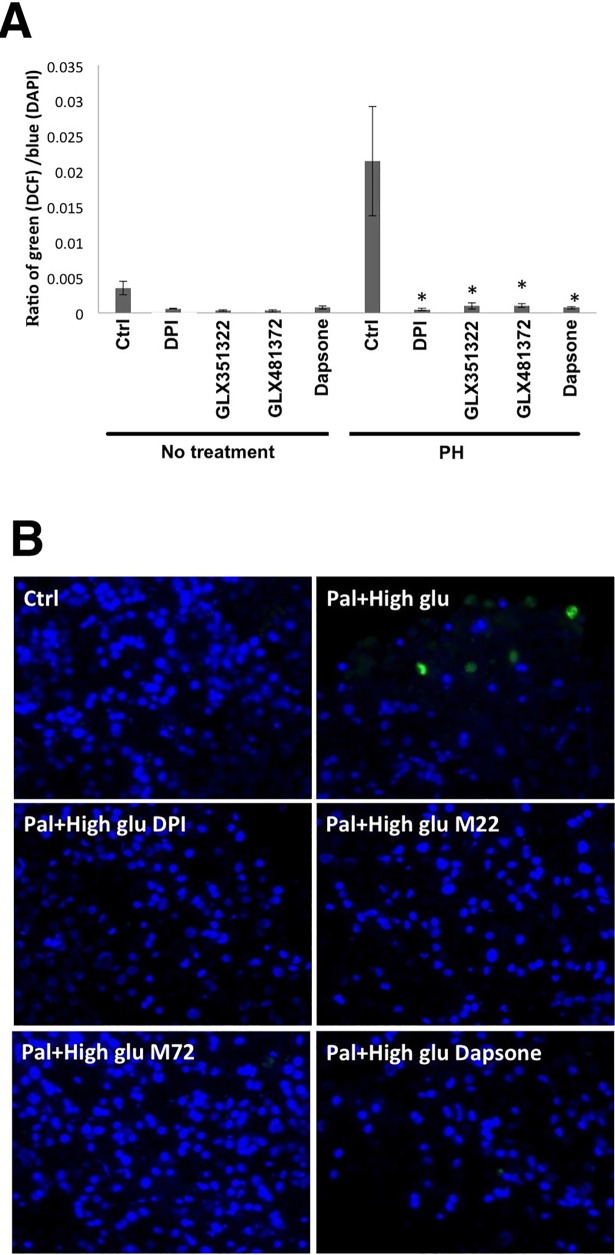
ROS production in human islet cells. (A) Quantification of ratio of DCF fluorescence to DAPI fluorescence intensity. Human islets were incubated at control condition (Ctrl) or with palmitate (1.5 mM + 2% BSA) + high glucose (20 mM) (PH) for 24h. During the last 3h of the 24h incubation Nox inhibitors: DPI (0.2 μM), Dapsone (10 μM), GLX351322 (10 μM) and GLX481372 (6 μM) were added to the culture medium. each treatment group, 5 images (100–250 cells in each image) were taken. The intensities of green (DCF) and blue (DAPI) signals were quantified by Image J software. Results are means ± S.E.M for 4 donors. * denotes P<0.05 compared with PH using repeated measurements one-way ANOVA and Fisher LPD. For (B) Representative photographs showing DCF and DAPI fluorescence. M22 depicts GLX351322 and M72 depicts GLX481372.

**Fig 4:**
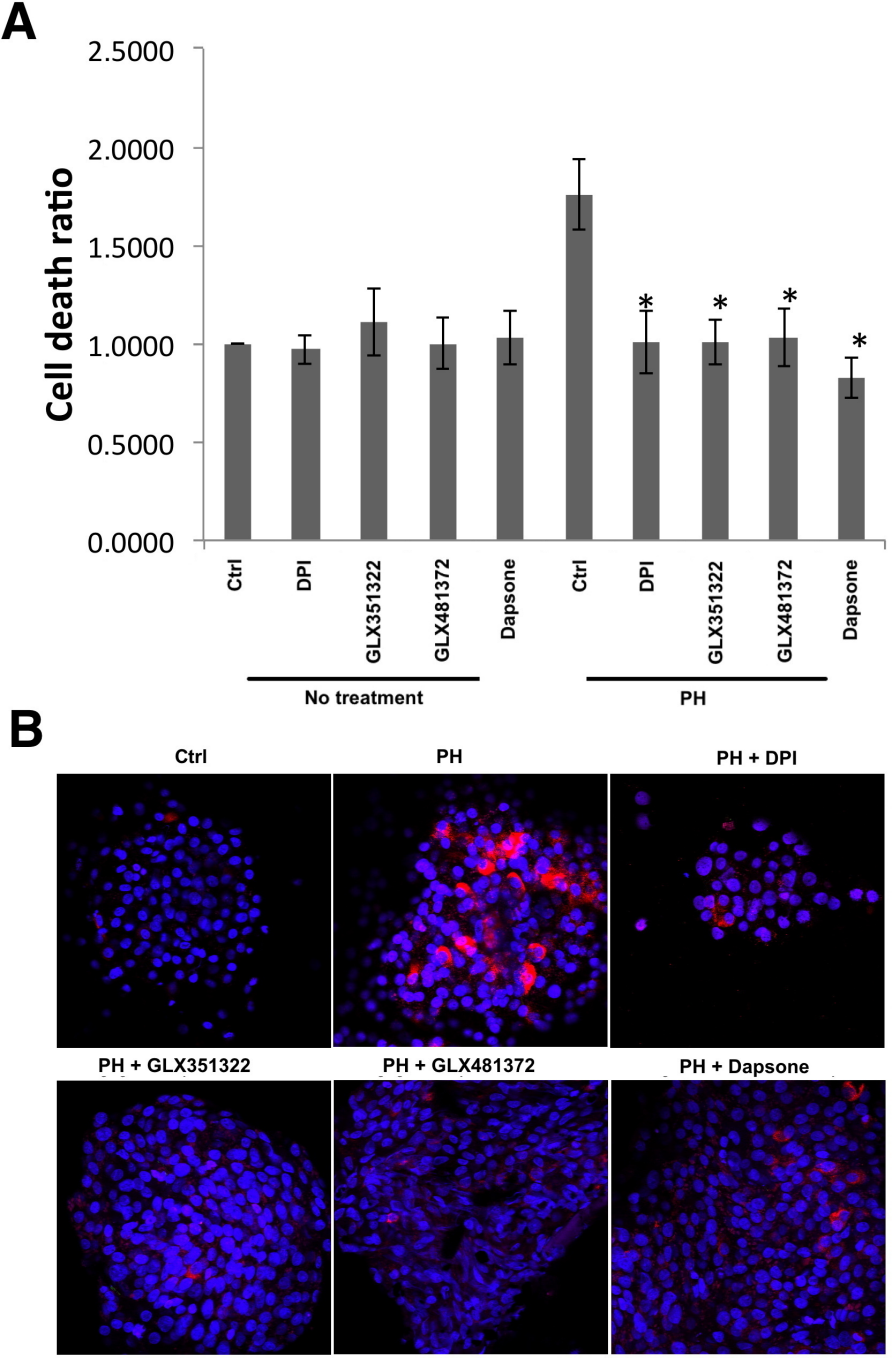
Effects of DPI, GLX351322, GLX481372 and Dapsone on human islet cell viability and caspase 3 activation. (A) Quantification of relative cell death (ratio PI/Bisbenzimide). Human islets were incubated at control conditions or with palmitate (1.5 mM + 2% BSA) + high glucose (20 mM) (PH) for 24h with Nox inhibitors: DPI, Dapsone, GLX351322 and GLX481372 (same concentrations as described in Fig 3 and present during the entire 24h exposure period). For each treatment group, 5 images (100–250 cells in each image) were taken. Islets were photographed in an inverted fluorescence microscope and the intensities of red (PI) and blue (Bisbenzimide) signals were quantified using Image J software. Results are means ± S.E.M for 7 human islet donors. * denotes P<0.01 compared with PH using repeated measurements one-way ANOVA and Fisher PLD. (B) Representative images of cleaved caspase 3 staining (red) after a 24h palmitate + high glucose incubation with and without Nox inhibitors.

The Nox inhibitor DPI is a pan-Nox inhibitor with IC_50_ values for all Nox enzymes in the nM range [25]. Dapsone has been reported to inhibit the expression /activity of both Nox4 and DUOX1 at μM concentrations [26,27], but its effects on other Nox enzymes are not known. GLX351322 and GLX481372 both inhibit Nox4, but also other Nox isoforms (Table 1). DPI, dapsone, GLX351322 and GLX481372 all reduced human islet ROS production during the last 3h of a 24h high glucose + palmitate exposure (Fig 3). This was paralleled by improved viability as indicated by a decreased PI and Bisbenzimide ratio and a reduced activation of caspase 3 (Fig 4).

**Table 1:** Summary of Nox inhibitor characteristics.

	Nox4 IC_50_ μM	Nox1 IC_50_ μM	Nox2 IC_50_ μM	Nox3 IC_50_ μM	Nox5 IC_50_ μM	GO inhibition μM	XO inhibition μM	Endogenous redox activity (DPPH)
DPI [26]	0.1	0.2	0.1		0.02		Yes	Not performed
Phox-I2 [13]	Inactive (Phox-I1)		1.0				Inactive	No
ML171 [11]	5	0.25	5				5.5	
GLX351322 [12]	5		40				Inactive	No
GLX481372	0.68	7	16	3.2(a)	0.6		Inactive	No
GLX7013114	0.3	Inactive	Inactive	Inactive	Inactive	Inactive	Inactive	No

Personal communication Dr. Vincent Jaquet, Centre Médical Universitaire, Geneva, Switzerland (a)

### Effects of selective Nox1, Nox2 and Nox4 inhibitors on human islet cell viability

**Fig 5:**
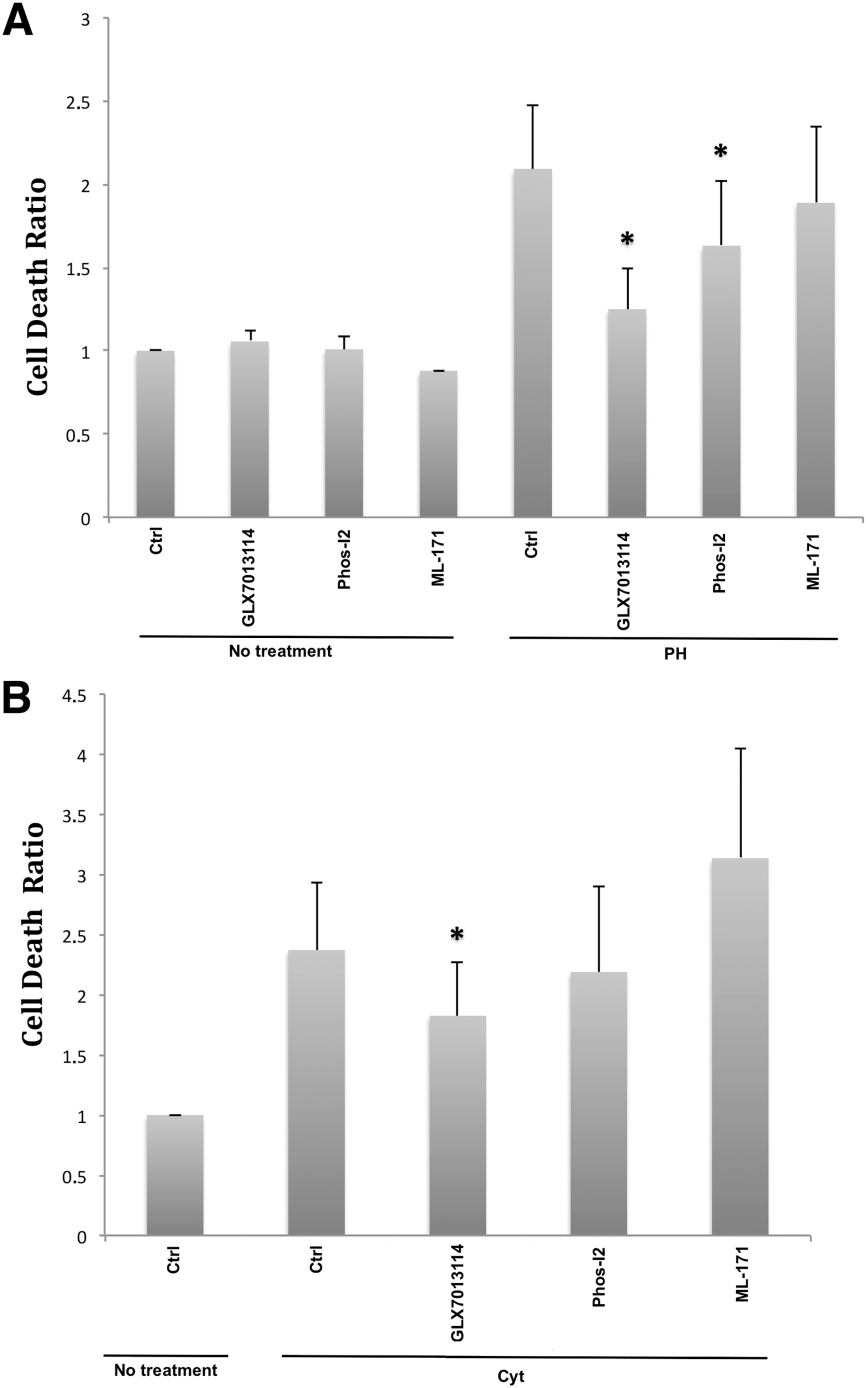
Effects of ML-171, Phos-I2 and GLX7013114 on human islet cell viability. Human islets were incubated at control condition, with IL-1**β** (20 ng/ml) + IFN-**γ** (20 ng/ml) (A), or with palmitate (1.5 mM + 2% BSA) + high glucose (20 mM) (PH) (B) for 2 days with or without Nox1 inhibitor ML-171 (2 **μ**M), Nox2 inhibitor Phos-I2 (2 **μ**M) and Nox4 inhibitor GLX7013114 (1 **μ**M). Islets were photographed in an inverted fluorescence microscope and the intensities of red (PI) and blue (Bisbenzimide) signals were quantified using Image J software. Results are means ± S.E.M for 7 human islet donors. * denotes P<0.05 compared with cyt or PH, respectively, using repeated measurements one-way ANOVA and Fishers PLD post-hoc test.

We used inhibitors targeting specific Nox isoforms to assess the contributions of specific Nox isoforms in cytokine and palmitate + high glucose induced human islets cell death. The Nox1 selective inhibitor ML171 [11], at a concentration of 2 μM, failed to reduce human islet cell death during a 48h culture period with cytokines or high glucose + palmitate (Fig 5). The Rac1/Nox2 selective inhibitor Phox-I2 [13], at a concentration of 2 μM, also failed to protect human islets against cytokines but partially protected against high glucose + palmitate. On the other hand, the highly selective Nox4 inhibitor GLX7013114, at a concentration of 1 μM, protected both against cytokines and high glucose + palmitate (Fig 5).

### Effects of the Nox4 inhibitor GLX7013114 on EndoC- βH1 cell ROS production and viability

**Fig 6:**
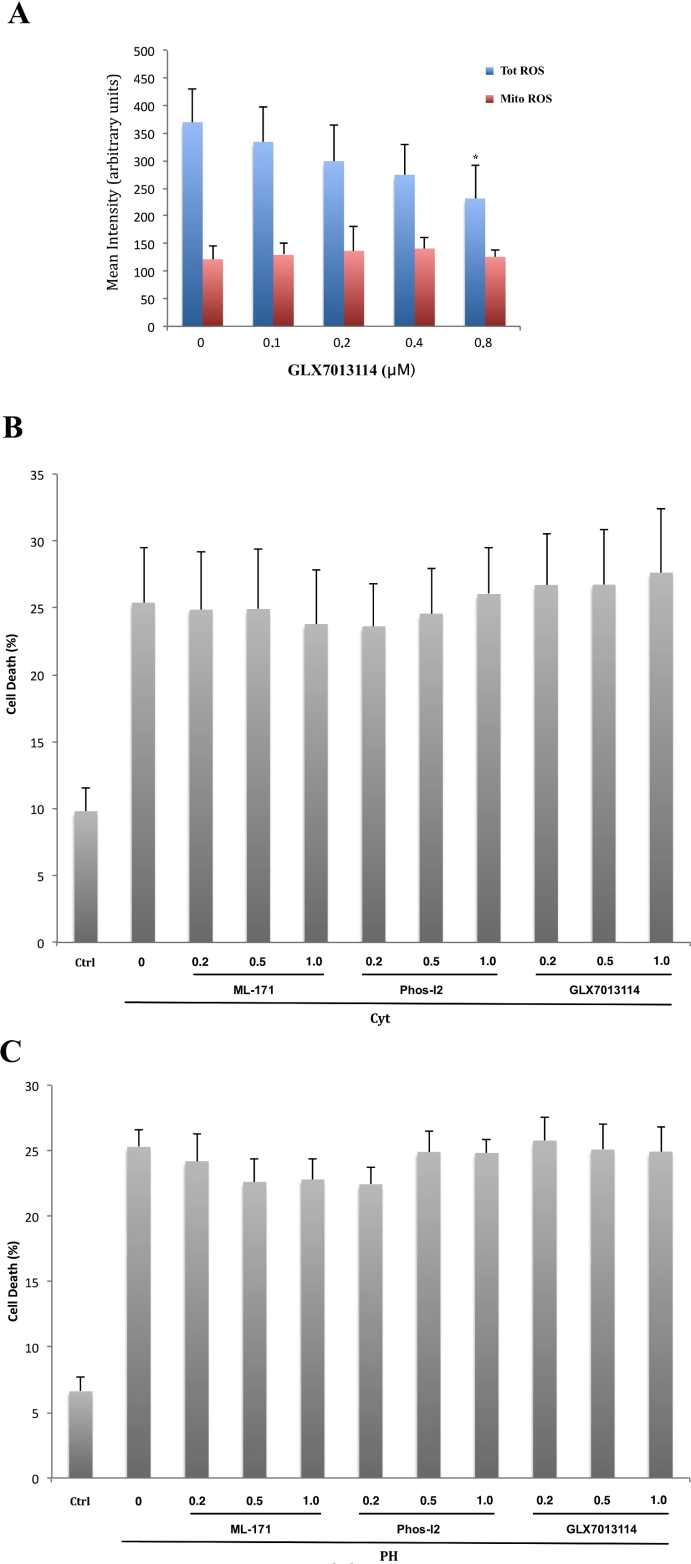
Effects of ML-171, Phos-I2 and GLX7013114 on EndoC-βH1 cell ROS production and viability. (A) Flow cytometry quantification of DCF (total ROS) and MitoSOX (mitoROS) intensities at control conditions. Results are means ± S.E.M for 3 independent experiments. * denotes P<0.05 compared with control using Student’s paired t-test. (B) Flow cytometry quantification of cell death after a 2-day incubation with cytokines (cyt) supplemented with 0.2 μM, 0.5 μM or 1.0 μM of Nox1 inhibitor ML-171, Nox2 inhibitor Phos-I2 or Nox4 inhibitor GLX7013114, respectively. Results are means ± S.E.M for 7 independent experiments. (C) Flow cytometry quantification of cell death after 24h incubation with palmitate (1.5 mM + 2% BSA) + high glucose (20 mM) (PH) supplemented with 0.2 μM, 0.5 μM or 1.0 μM of Nox1 inhibitor ML-171, Nox2 inhibitor Phos-I2 or Nox4 inhibitor GLX7013114, respectively. Results are means ± S.E.M for 8 independent experiments.

As inhibition of Nox4 both counteracted cytokine and palmitate + high glucose induced human islet cell death, we next investigated whether this occurs also in a population of pure beta-cells, the beta-cell line EndoC- βH1. Using H_2_DCFDA and MitoSOX Red probes, ROS production of EndoC- βH1 cells was analyzed. We observed that GLX7013114 reduced total ROS production, but not mitochondrial superoxide production, in EndoC- βH1 cells in a concentration dependent manner (Fig 6A). Despite this, the three Nox inhibitors, ML171 (Nox1) Phox-I2 (Nox2) and GLX7013114 (Nox4), at concentrations up to 1 μM, all failed to affect cell death rates in EndoC- βH1 cells exposed to cytokines (Fig 6B) or high glucose + palmitate (Fig 6C).

## Discussion

In collaboration with Glucox Biotech AB (GB), we presently report the development of two novel Nox inhibitors, of which one (GLX7013114) is highly selective in the inhibition of Nox4. To our knowledge GLX7013114 is the first reported Nox inhibitor that is highly selective for Nox4. It is known that all Nox isoforms contain the same core (gp91-Phox and gp22-Phox) catalytic domain that generates superoxide. Many Nox-inhibitors available today, including DPI and probably also GKT136901, inhibit this common gp91-Phox/gp22-Phox catalytic site, which explains the lack of total selectivity for most Nox inhibitors. In the case of GLX7013114 we believe that this inhibitor targets a unique “Nox4 hot-spot” that selectively inhibits the Nox4 enzyme distant from the common gp91-Phox/gp22-Phox catalytic site, thereby providing total selectivity towards other Nox isoforms.

The two novel Nox inhibitors, GLX481372 and GLX7013114, were presently compared with previously characterized Nox inhibitors with regard to their ability to prevent human islet cell death provoked by cytokines or high glucose + palmitate. We did not analyze effects of the Nox inhibitors on insulin release, and we can therefore only draw conclusions when it comes to beta-cell death, and not beta-cell function. GLX481372, which selectively inhibits Nox4 and Nox5, was compared with the pan-Nox inhibitor DPI [11], the Nox4 and DUOX1 inhibitor dapsone [26,27] and the Nox4 and Nox1 inhibitor GLX351322 [13]. All four inhibitors ameliorated high glucose + palmitate-induced ROS production and cell death, suggesting that at least one of the different Nox enzymes participates in metabolic stress-induced beta-cell death. This fits well with our previous study in which GLX351322 protected against high glucose-induced inhibition of human islet insulin release and survival [12]. At the time of our previous publication we had only information on GLX351322 selectivity for Nox4 over Nox2, but since then additional experimentation has revealed that this compound inhibits Nox1 and Nox5 with similar efficacy as Nox4 (personal communication; Pamela Kleikers, Maastricht University, NL). Thus, none of the four Nox inhibitors are sufficiently selective to pinpoint a specific Nox enzyme, but it is noteworthy that all four compounds inhibit Nox4 at relatively low concentrations.

In a subsequent series of experiments, we compared also GLX7013114, which inhibits Nox4 only and already at sub-micromolar concentrations, with the selective Nox1 inhibitor ML171 and the selective Rac1/Nox2 inhibitor Phox-I2. In these experiments we observed that ML171 failed to protect human islets from cell death, whereas both Phox-I2 and GLX7013114 protected against high glucose + palmitate. The cell death response to cytokines was counteracted by GLX7013114, but not by Phox-I2 and ML171. These findings support the notion that Nox4 participates in both cytokine- and high glucose + palmitate-induced cell death, whereas Nox2 may play a role only in metabolic stress. According to a recent publication, however, Nox2 gene knock-out did not protect against high glucose-induced beta-cell death in C57BL/6J mice [28]. The reason for this inconsistency is not clear, but it may be that human islets respond differently to Nox inhibition as compared to mouse islets. Alternatively, pharmacological inhibition of an enzyme may evoke effects distinct from those associated with a gene deletion. Indeed, pharmacological inhibition does not block the activity of an enzyme completely, and a partial loss of Nox enzyme activity may be more pertinent to an *in vivo* situation where attenuation of peak activity is desired without loss of basal ROS production, as this will reduce the risk for unwanted side effects. It has been proposed that Nox1, rather than Nox4, participates in HG + palmitate and cytokine-induced beta-cell dysfunction and death [16]. In these experiments, however, Nox inhibitors were used that do not differ in selectivity for Nox4 and Nox1.

The notion that Nox4-mediated ROS production participates in islet cell death was further strengthened by our finding that the Nox4 inhibitor GLX7013114 mimicked the protective effects observed using the other Nox4 inhibitors dapsone, GLX351322 and GLX481372. Unfortunately, the IC_50_ for GLX7013114-induced DUOX1/2 inhibition is not known, which is the case for most other Nox inhibitors as well. Thus, the possibility that Nox4 inhibitors affect also DUOX1/2, or some other unknown target, cannot be excluded.

Expression of the Nox4 enzyme, albeit at a low level, could be detected in both human islets, which consist of not only beta-cells but also other islet cell types, and in the human beta-cell line EndoC-βH1. In human islet cells Nox4 subcellular localization varied among islet cells and in some cells there were signs of accumulation at the nuclear membrane. In EndoC-βH1 cells Nox4 was rather uniformly distributed with the exception of late mitotic cells, in which there were signs of cytoplasmic Nox4 accumulation. This observation concurs well with recent reports describing a role of Nox4 in beta-cell differentiation/proliferation [29,30] and that Nox4 activity prevents senescence [31]. However, as cytokines failed to affect expression or redistribute Nox4 in EndoC-βH1 cells, it is unlikely that Nox4 activity is controlled via dramatic changes in levels or subcellular localization, and that its activation instead may result from posttranslational modifications. Indeed, it has been observed that Nox4 is controlled not only by expression, but also via direct binding to ATP [32]. Interestingly, both high glucose + palmitate and cytokines reduce beta-cell ATP levels [33,34], which might underlie Nox4 activation and increased ROS production. Nox4 is also controlled via protein-to-protein interactions [35]. For example, Nox4 protein activity is modulated via binding to p22phox, PDI, Pldip2, Tsk4/5 and Hsp70 [35], and such protein-to-protein interactions may in turn be controlled by different phosphorylation events. In line with this, it has been demonstrated that Nox2 becomes activated in response to Src tyrosine kinase substrate phosphorylation [36]. It has also been reported that mesenchymal cell activation and migration may involve Src-mediated Nox4 activation [37] and that Nox4 mediates Src-dependent PDK1 phosphorylation [38]. Furthermore, it has recently been proposed that Nox4 stimulates carnitine-palmitoyl transferase-1-induced fatty acid oxidation leading to NLRP3 inflammasome activation [39]. Thus, it is tempting to speculate that high glucose + palmitate/cytokines in islet cells, possibly via a decrease in ATP and/or tyrosine phosphorylation events, promotes Nox4-mediated ROS production, inflammasome-initiated inflammation, beta-cell dysfunction and an impaired glucose tolerance.

In adipocytes and in early stages of T2DM, Nox4 activity may be stimulated by hyperglycemia via an increased flow through the pentose phosphate shuttle and augmented NADPH, and by palmitate via a TLR4-dependent mechanism [40]. These early events are thought to precede and/or initiate ROS production by other Nox enzymes and from the mitochondrial respiratory chain [40]. Also in other situations, for example during angiogenesis, an initial Nox4 activation triggers Nox2 to further stimulate ROS production from the mitochondrial respiratory chain [41]. Such a dual Nox-mediated activation mechanism might explain why both GLX7013114 and Phox-I2 presently protected against high glucose + palmitate-induced islet cell death.

Interestingly, even though ROS production was suppressed, the Nox4 inhibitor GLX7013114 failed to protect against cytokine- and high glucose + palmitate-induced EndoC-βH1 cell death. This can either be taken to indicate that the toxic effects of Nox4 requires paracrine interactions between different islet cell types, for example with endothelial cells [42], or that the EndoC-βH1 cell production of ROS/resistance to ROS is different from that in primary human islet beta-cells. In either case, it is likely that EndoC-βH1 cells are not suitable for studies of Nox4-induced beta cell death.

In summary, we report the generation of the novel Nox4 selective inhibitor GLX7013114 and that this compound protects islet cells against both high glucose + palmitate- and cytokine-induced death. Furthermore, as Nox4 expression or subcellular localization was not affected in response to metabolic or inflammatory stress, it is likely that Nox4 activity is controlled post-translationally. We propose that selective Nox4-inhibition may be a therapeutic strategy in type 2 diabetes.

## Supporting information

S1 Table Characteristics of GLX481372. (DOCX)

S2 Table Characteristics of GLX7013114. Personal communication with Freddy Heitz (Head of Screening and Biotechnology Genkyotex S A, Switzerland) *; In silico determination **.
(DOCX)

S3 Table Characteristics of Nox expressing cells. (DOCX)

S1 Fig Structure of GLX481372. (TIF)

S2 Fig Increased concentration of GLX7013114 inhibitor titrates with a decrease of hydrogen peroxide production from Nox4 expressing CJ HEK 293 cells. Decreasing concentrations (200–0.003 μM) GLX7013114 and GKT136901 that were incubated in an 11-step 1/3 dilution in a 96 well plate with Nox4 expressing CJ HEK 293 cells. Amplex Red was used as probe to measure hydrogen peroxide production.
(JPG)

S3 Fig GLX7013114 does not inhibit Nox5 enzymatic activity in HEK 293 cell overexpressing Nox5. Decreasing concentrations (200–0.01 μM) GLX7013114, GKT136901 and DPI was incubated in an 11-step 1/3 dilution in a 96 well plate with Nox4 expressing CJ HEK 293 cells. Amplex Red was used as probe to measure hydrogen peroxide production.
(JPG)

S4 Fig GLX7013114 does not affect DPPH absorbance. DPPH was incubated with decreasing concentrations (200–0.003 μM) of GLX7013114 or GKT136901 (positive control) and absorbance at 518 nm was measured after 60 min.
(JPG)

S5 Fig GLX7013114 does not inhibit Xanthine oxidase activity. The enzyme was incubated with decreasing concentrations (200–0.003 μM) of GLX7013114 and GKT136901 and DPI as positive control and with Amplex Red analysis as read out.
(JPG)
