# Supplementary material for: The novel NADPH oxidase 4 selective inhibitor GLX7013114 counteracts human islet cell death in vitro
Source: PLoS One. 2018 Sep 28;13(9):e0204271. doi: 10.1371/journal.pone.0204271 (PMC6161897; doi:10.1371/journal.pone.0204271)
Supplement: S1 Table — (DOCX) [file pone.0204271.s001.docx]

| - IC_50_, Nox4 inhibition in TRex Nox4 HEK 293 (Amplex Red analysis) | - 0.68 μM |
| --- | --- |
| - IC_50_, Nox2 inhibition in hPBMC cells | - 16 μM |
| - IC_50_, Nox1 inhibition in CHO (Amplex Red Glucox analysis) | - 7 μM |
| - IC_50_, Nox5 inhibition in Nox5 HEK 293 (“Vincent and Tamara” analysis) | - 0.57 μM |
| - Solubility, in KP from solid substance | - 2.7 and 2.5 μM (isotonic) |
| - Kinetic solubility in silico | - 6 μM |
| - Kinetic solubility in vitro | - >100 μM |
| - Permeability, Caco-2 cells, Papp a-b | - (12+/- 1.6) X 10^-6^ cm/s |
| - Permeability, Caco-2 cells, Papp b-a | - (43+/- 5.8) X 10^-6^ cm/s |
| - Chemical stability pH 2, 7.4 and 10 for 20h in room temp | - No pH dependent instability |
| - Human plasma protein binding/ stability | - 0.07%/107% |
| - Human/mouse metabolic stability | - 27min/36min |
