# Supplementary material for: The novel NADPH oxidase 4 selective inhibitor GLX7013114 counteracts human islet cell death in vitro
Source: PLoS One. 2018 Sep 28;13(9):e0204271. doi: 10.1371/journal.pone.0204271 (PMC6161897; doi:10.1371/journal.pone.0204271)
Supplement: S2 Table — Personal communication with Freddy Heitz (Head of Screening and Biotechnology Genkyotex S A, Switzerland) *; In silico determination **. (DOCX) [file pone.0204271.s002.docx]

| - IC50, Nox4 inhibition in CJ Nox4 HEK 293 using Amplex Red analysis as detection probe | - 0.3 μM |
| --- | --- |
| - IC50, Nox4 inhibition in membrane based Nox4 activity using Amplex Red analysis as detection probe | - 0.56 μM* |
| - IC50, Nox4 inhibition in membrane based Nox4 activity using CBA analysis as detection probe | - 0.52 μM* |
| - IC50, Nox1 inhibition in CHO cells using Amplex Red analysis as detection probe | - Inactive |
| - IC50, Nox2 inhibition in human neutrophils using Luminol analysis as detection probe | - Inactive |
| - IC50, Nox5 inhibition in Nox5 HEK 293 using Amplex Red analysis as detection probe | - Inactive |
| - IC50, Nox1, Nox2, Nox3, Nox5 inhibition in whole cells, CHO and HEK 293 (Amplex Red analysis)* | - All Inactive* |
| - IC50, Nox1, Nox2, Nox3, Nox5 inhibition in membrane assay of CHO and HEK 293 (Amplex Red) | - All Inactive* |
| - IC50, Glucose oxidase | - Inactive * |
| - IC50, Xanthine oxidase | - Inactive |
| - Oxygen consumption in Nox1 CHO cells | - No inhibition* |
| - Oxygen consumption in Nox4 HEK cells | - Inhibition of Nox4 dependent oxygen consumption* |
| - Kinetic solubility in vitro | - 1.1 μM* |
| - Chemical stability dissolved in DMSO for more than 2 month in room temp. | - 100 % |
| - Solubility in silico | - 5 μM** |
| - Metabolic stability - In human liver microsomes half-life | - 1 min and 2.5 min* |
| - Permeability, Caco-2 cells, Papp a-b | - 53 x 10^-6^ cm/s ** |
| - Pharmacokinetics, i.p. mouse t1/2 | - 0.4 h |
