# Supplementary material for: The novel NADPH oxidase 4 selective inhibitor GLX7013114 counteracts human islet cell death in vitro
Source: PLoS One. 2018 Sep 28;13(9):e0204271. doi: 10.1371/journal.pone.0204271 (PMC6161897; doi:10.1371/journal.pone.0204271)
Supplement: S3 Table — (DOCX) [file pone.0204271.s003.docx]

| - Nox enzyme | - Cell line | - Expressed subunits | - Selection or activation agent | - Detection assay |
| --- | --- | --- | --- | --- |
| - Nox4 | - CJ HEK 293 with Nox4 constitutively expressed heterologous expression | - Nox4 | - Neomycin | - Amplex red |
| - Nox4 | - HEK 293 TRex heterologous expression | - Nox4 | - Tetracyclin (inducing expression of Nox4) | - Amplex red |
| - Nox1 | - CHO/heterologous expression | - Nox1, CYBA, Noxo1, NoxA1 | - PMA | - Luminol or Amplex red |
| - Nox5 | - HEK293/ heterologous expression | - Nox5 | - Ionomycin and PMA | - Amplex red |
| - Nox2 | - PLB-985/natural expression after differentiation with DMSO | - Nox2 | - PMA | - Luminol |
