# Supplementary figures and images for: The novel NADPH oxidase 4 selective inhibitor GLX7013114 counteracts human islet cell death in vitro
Source: PLoS One. 2018 Sep 28;13(9):e0204271. doi: 10.1371/journal.pone.0204271 (PMC6161897; doi:10.1371/journal.pone.0204271)

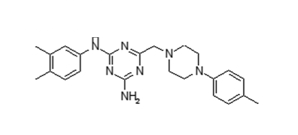

Supplement: S1 Fig — (TIF) [file pone.0204271.s004.tif]

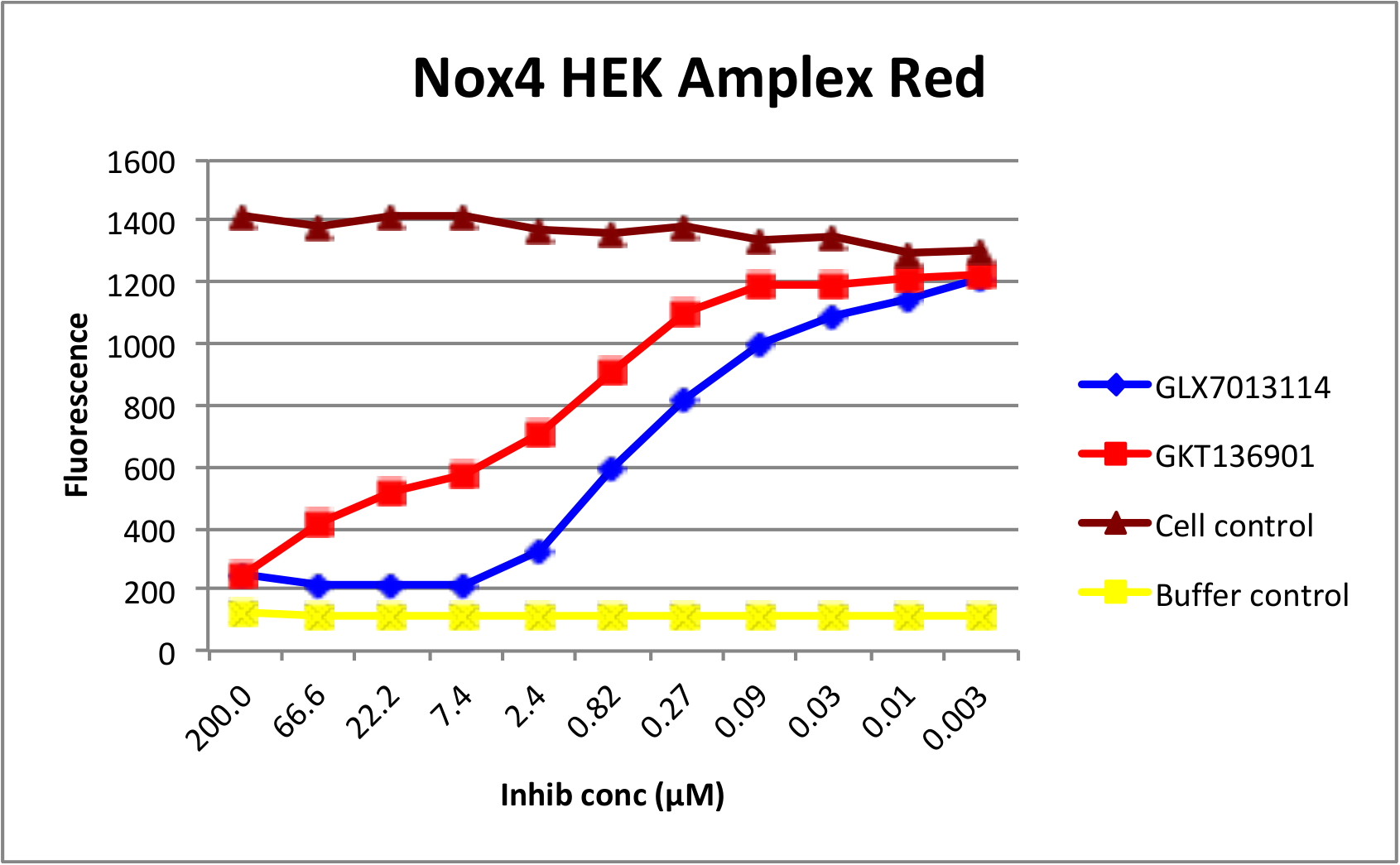

Supplement: S2 Fig — Decreasing concentrations (200–0.003 μM) GLX7013114 and GKT136901 that were incubated in an 11-step 1/3 dilution in a 96 well plate with Nox4 expressing CJ HEK 293 cells. Amplex Red was used as probe to measure hydrogen peroxide production. (JPG) [file pone.0204271.s005.jpg]

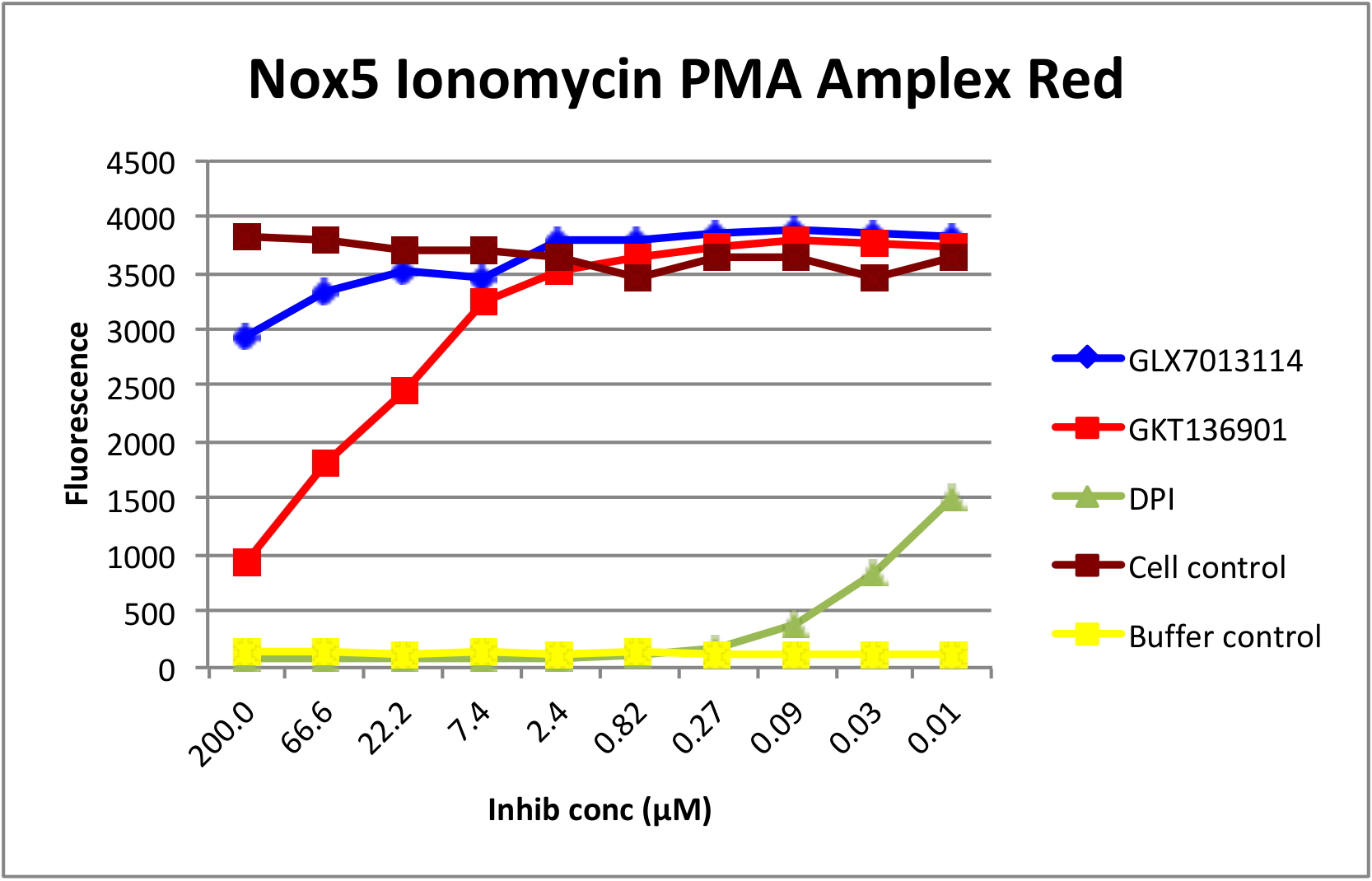

Supplement: S3 Fig — Decreasing concentrations (200–0.01 μM) GLX7013114, GKT136901 and DPI was incubated in an 11-step 1/3 dilution in a 96 well plate with Nox4 expressing CJ HEK 293 cells. Amplex Red was used as probe to measure hydrogen peroxide production. (JPG) [file pone.0204271.s006.jpg]

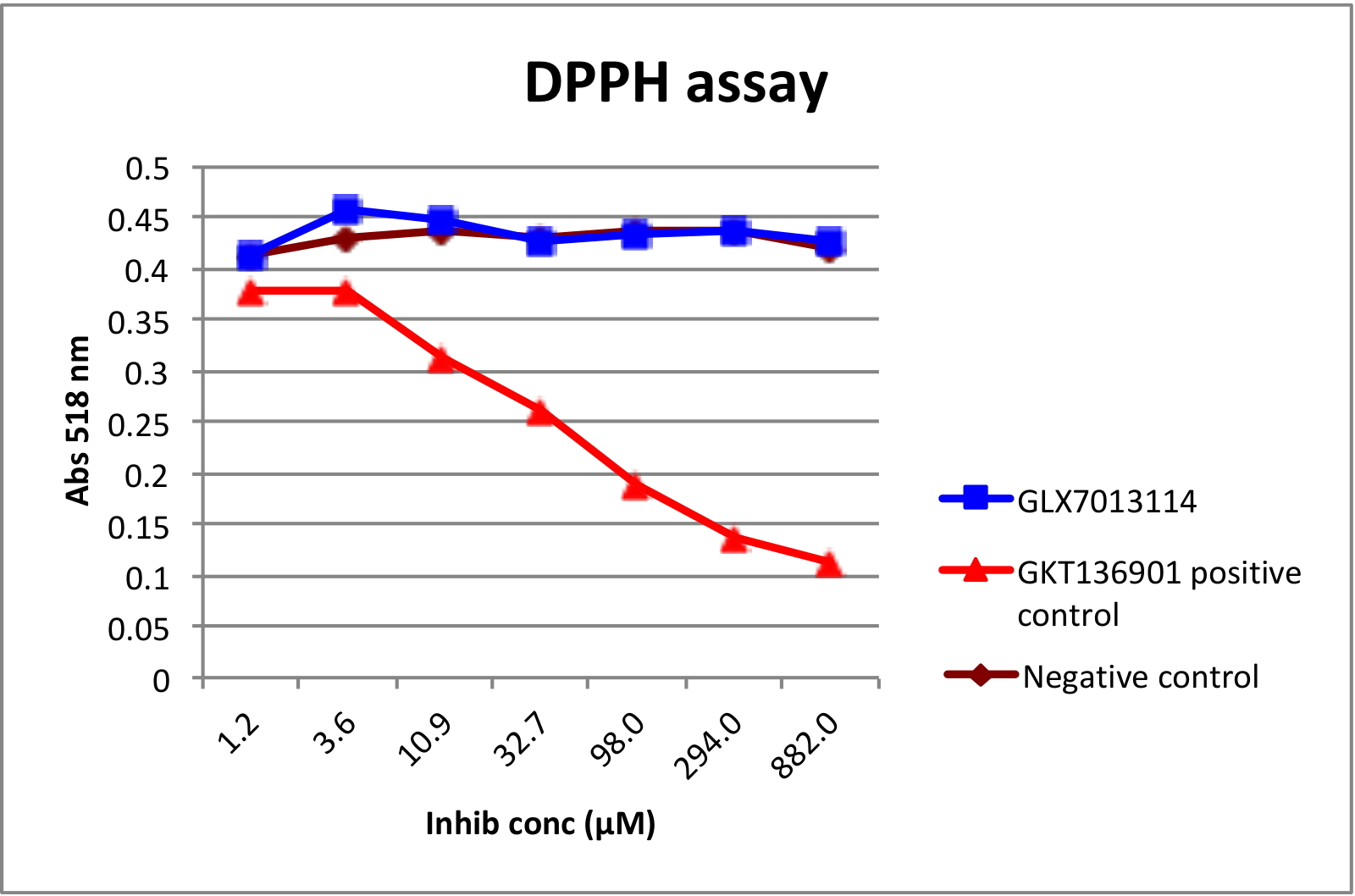

Supplement: S4 Fig — DPPH was incubated with decreasing concentrations (200–0.003 μM) of GLX7013114 or GKT136901 (positive control) and absorbance at 518 nm was measured after 60 min. (JPG) [file pone.0204271.s007.jpg]

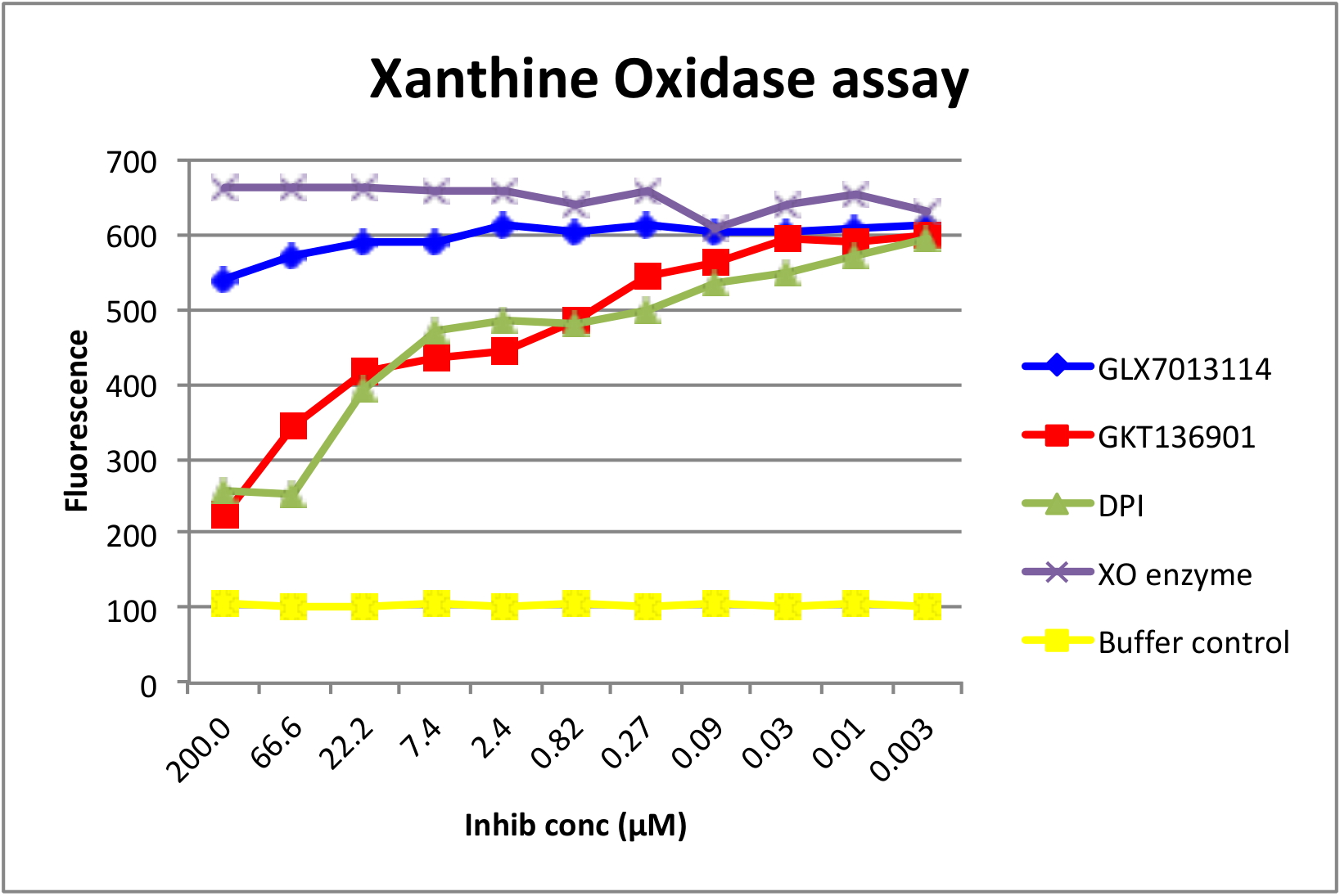

Supplement: S5 Fig — The enzyme was incubated with decreasing concentrations (200–0.003 μM) of GLX7013114 and GKT136901 and DPI as positive control and with Amplex Red analysis as read out. (JPG) [file pone.0204271.s008.jpg]
